# WeCareAdvisor, an Online Platform to Help Family Caregivers Manage Dementia-Related Behavioral Symptoms: an Efficacy Trial in the Time of COVID-19

**DOI:** 10.1007/s41347-021-00204-8

**Published:** 2021-03-25

**Authors:** Laura N. Gitlin, Nicole Bouranis, Vince Kern, Sokha Koeuth, Katherine A. Marx, Leslie A. McClure, Constantine G. Lyketsos, Helen C. Kales

**Affiliations:** 1 Drexel University, Philadelphia, PA USA; 2 University of California, Davis, CA USA; 3 Johns Hopkins University, Baltimore, MD USA

**Keywords:** Dementia, Caregiving, Technology, Behavioral and psychological symptoms of dementia, Online program

## Abstract

Dementia-related behavioral and psychology symptoms (BPSD) are undertreated and have negative consequences. However, families do not have access to disease information, tailored problem-solving  and effective management strategies, and with COVID-19, are more socially isolated and distressed. To address this dementia care gap, we describe a Phase III efficacy trial testing an online platform, WeCareAdvisor, and design modifications necessitated by COVID-19. WeCareAdvisor provides caregivers with disease information, daily tips, and a systematic approach for *describing* behaviors, *investigating* underlying causes, *creating* tailored strategies, and *evaluating* their effectiveness (DICE). The trial will enroll 326 caregivers nationwide, randomly assign them to immediately receive WeCareAdvisor (treatment), or a 3-month waitlist (control) and evaluate short (1- and 3-month) and long-term (6-month) outcomes for caregiver distress with and confidence managing BPSD, and BPSD occurrences. We will also evaluate utilization patterns with different prompting conditions: high-intensity (telephone and email reminders), low-intensity (email reminders), or no reminders to use WeCareAdvisor. COVID-19 necessitated design modifications resulting in greater inclusivity of caregivers from diverse races, ethnicities, and geographic areas. Key modifications include shifting from in-home, in-person interviewing to telephone; adjusting tool functionality from operating on a grant-funded iPad to caregivers’ personal internet-capable devices; and expanding recruitment from one metropolitan area to nationwide. Study modifications necessitated by COVID-19 facilitate national outreach, easier tool adoption, and enable more diverse caregivers to participate. This study addresses a critical dementia care need, and design modifications may shorten timeline from efficacy testing to commercialization.

Close to six million people in the USA have dementia, with most living at home and cared for by over 16 million family caregivers (Alzheimer’s Association, 2020). Behavioral and psychological symptoms of dementia (BPSD) dominate disease presentation. BPSD occur across disease etiologies and stages and have been shown to occur in neuropsychiatric syndromes identified as depression, apathy, psychosis, agitation, sleep disturbances, or executive dysfunction (Kales et al., 2014; Lawlor, 2002). Other behaviors (shadowing, rejection of care) may not fall within formal syndromes but are equally problematic to families (Regier et al., 2020).

BPSD have wide ranging deleterious consequences for people living with dementia (PLWD). These include poor quality of life, excess morbidity, hospitalization, nursing home placement, and mortality. Untreated, clinically significant BPSD lead to more rapid conversion from mild cognitive impairment to dementia and quicker disease progression (Forrester et al., 2016; Sacuiu et al., 2016; Hodgson et al., 2014).

For families, managing BPSD is one of the most challenging aspects of care, causing intense burden and upset, and posing threats to their own health and well-being (Van Den Winjgaart et al., 2007). Caregivers of PLWD with BPSD are more distressed and depressed than those not managing BPSD (De Vugt et al., 2004). Caregivers frequently manage multiple behavioral disturbances simultaneously, with the occurrence of four or more BPSD associated with clinically significant depression and burden (Arthur et al., 2017). Caregivers may also experience reduced employment and income, more time spent caregiving, and higher out-of-pocket costs (Jutkowitz et al., 2017). Approximately 30% of dementia care costs are directly attributable to managing BPSD (Beeri et al., 2002).

Although a common clinical feature, BPSD remain under-detected and undertreated (Gitlin et al., 2012). Presently, there are no FDA approved medications for treating BPSD. Typically, psychiatric medications (e.g., antipsychotics, anticonvulsants) are prescribed. While antipsychotics have the greatest proven efficacy for BPSD in research trials, they demonstrate only modest benefits and effect sizes (Schneider et al., 2006), and are associated with significant risks including mortality. This has resulted in FDA black box warnings regarding their use for this purpose (Kales et al., 2012; Maust et al., 2015). Medications like anticonvulsants have been used as alternatives to antipsychotics; however, they have similar risks and less evidence of benefit. In contrast, nonpharmacologic strategies are recognized as first-line treatment, except in emergency situations, when behaviors could lead to imminent danger (Rabins et al., 2006; Salzman et al., 2008).

Thus, families are typically left on their own to address symptoms with limited access to vetted knowledge and evidence-informed management strategies. This is particularly the case for families living in rural areas and of diverse races and ethnicities (Black et al., 2013; Werntz et al., 2015). COVID-19 has intensified the challenges of managing BPSD. With the need to stay at home and limit social contact, caregivers report managing more BPSD, and greater confusion and boredom in PLWD. Also, with the closing of community-based supportive programs such as adult day, PLWD lack access to meaningful activities. In turn, caregivers are experiencing heightened distress, increased social isolation, and difficulty accessing supportive services and respite (UsAgainstAlzheimer’s, 2020).

The pandemic, however, has also highlighted the potential role of technology in supporting family caregivers. For example, most healthcare appointments and support groups are being offered and reimbursed through virtual formats (Cuffalo et al., 2020). Additionally, more older adults now than previously are using videoconferencing to connect with friends and relatives with 31% using videoconferencing at least once per week (Malani et al., 2020). These conditions all support the potential utility of a web-based tool to help caregivers manage BPSD. Caregivers report being comfortable with internet-capable devices (computers, tablets, smartphones), using the internet to find resources, and believe on-demand tools could be beneficial to them and the person living with dementia (AARP, 2016; Czaja et al., 2006; Dang et al., 2004; Fox et al., 2013; National Alliance for Caregiving, 2011).

In response, we plan to test an online platform for caregivers, WeCareAdvisor, that provides disease education, daily tips, and a systematic, evidence-informed approach (DICE) for describing behaviors, investigating causes, creating a tailored treatment plan, and evaluating the effectiveness of strategies to manage symptoms. We previously described the conceptual frameworks informing WeCareAdvisor, the stepwise and iterative user-informed approach to its development, and initial results from a pilot randomized trial showing high utilization of each of its features, reduction in caregiver distress, and a trend towards fewer behavioral and psychological symptom occurrences (Gitlin et al., 2016; Kales et al., 2014, 2018). Findings demonstrated proof of concept, tool acceptability, and positive outcomes, and supported moving forward with a large efficacy trial. Also demonstrated was the need to understand differential utilization patterns and the type of prompt needed to motivate continued tool use.

The purpose of this article is to describe our protocol for a Phase III efficacy trial and highlight study modifications necessitated by methodological challenges posed by COVID-19. Our premise is that these design pivots strengthen study rigor and provide a unique opportunity to test WeCareAdvisor with a national sample. Our revised approach due to COVID-19 may shorten the timeframe from efficacy to commercialization and serve as a model for testing other online dementia care support programs for family caregivers.

## Study Overview

**Fig. 1:**
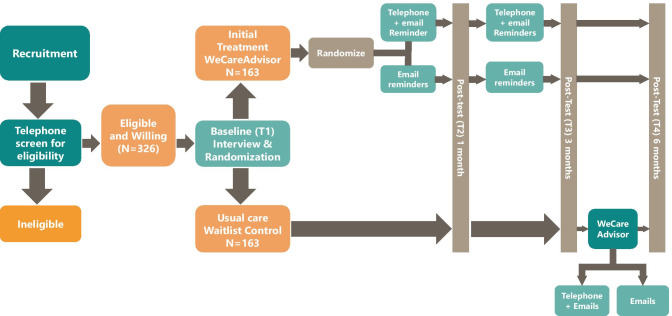
Study design for efficacy trial

The WeCareAdvisor is being tested in a Phase III randomized trial with 326 caregivers. As shown in Fig. 1, the trial will evaluate the effects of WeCareAdvisor on caregiver distress with and confidence managing BPSD, and also whether tool use results in reductions in BPSD (frequency × severity) in PLWD compared to a waitlist control group at 1 and 3 months. The trial will also evaluate whether different communication prompting strategies (high-intensity telephone plus email reminders, or low intensity automated email reminders) are effective in prompting tool use and impact outcomes. Also, we will examine whether the initial treatment group continues to use WeCareAdvisor between 3 and 6 months without receiving any prompts. Finally, at 3 months, the waitlist control group will receive access to WeCareAdvisor and the trial will evaluate whether this group has the same magnitude of benefit as the initial treatment group following their tool use between 3 and 6 months.

On an exploratory basis, the effects of WeCareAdvisor on other important clinical outcomes will be examined including psychotropic medication use and functional dependence, and theory-informed mediator and moderator (nonwhite/white; spouse/nonspouse) effects on 3- and 6-month outcomes and tool utilization. Lastly, study satisfaction and perceived benefits of tool use will be evaluated following the conclusion of a caregiver’s study participation.

### Specific Aims

This efficacy trial has four study aims. These are to (a) evaluate short-term (1 and 3 months) and (b) long-term (6-month) effects of WeCareAdvisor on caregiver distress, confidence managing BPSD, and BPSD (frequency and severity); (c) examine if the waitlist control group receives similar long-term benefits to the immediate group; and (d) determine tool utilization and outcomes under two different prompt types: low-intensity (automated email reminder) or high-intensity (telephone and email). The study hypothesis is that caregivers in the initial treatment group will report short- and long-term reduced distress, improved confidence and fewer BPSD compared to the waitlist group at 1 and 3 months. Also hypothesized is that the waitlist group will report similar benefits at 6 months as the initial treatment group after using WeCareAdvisor (study months 3 to 6). Finally, it is expected that both high-intensity and low-intensity prompt groups will derive similar benefits. Understanding effectiveness and intensity of prompting has important implications for advancing a commercialization and implementation strategy.

### General Procedures

For caregivers contacting the research team with interest in the study trial, procedures will be explained and if interested, they will participate in a brief telephone screen to determine eligibility. Caregivers who are eligible and willing to participate will be scheduled for a baseline telephone interview within 2 weeks. After completing the interview, a second research staff member (trainer) will randomize the caregiver and contact them to indicate group allocation (initial treatment or 3-month waitlist control) and their prompting condition (telephone plus email, or email only). The trainer will also instruct the caregiver to download the tool onto their preferred internet-capable device (computer, tablet, or smartphone), provide an orientation to WeCareAdvisor, and walk the caregiver through the DICE approach (described below) for a behavioral symptom they seek to address. Caregivers assigned to the waitlist condition will be informed of next steps and will not be granted access to the tool until 3 months later.

All caregivers will receive 1- and 3-month follow-up interviews by interviewers masked to their group allocation. At the conclusion of 3 months, the initial treatment group will be encouraged to continue to use WeCareAdvisor, but they will not receive further prompt reminders. For the waitlist group, the trainer will contact them following their 3-month interview and provide instructions in tool use. All caregivers will be reassessed at 6 months by interviewers who will be aware that caregivers had access to WeCareAdvisor but not their group assignment or prompting condition. Caregivers will be offered a $15 gift card for each completed interview (a total of $60 in gift cards for completion of 4 interviews) and materials about dementia/behaviors will be shared as caregivers will not have access to the tool at the conclusion of their study participation.

### Initial Treatment Group

Following the baseline interview, caregivers assigned to the initial treatment condition will receive (1) access to the online platform, (2) optional email account setup for those without prior email access, (3) an orientation to WeCareAdvisor involving initial set up and practice using one DICE session, and (4) a brief video explaining tool use. Caregivers will be guided through accessing the platform, creating an account and completing a brief set of background questions to inform identification of a peer tool navigator which provides written instructions, examples, and if the caregiver chooses, audio instructions. Trainers will guide caregivers through the questions posed by the tool and help caregivers identify the first behavioral symptom they seek to address through the DICE approach. Then, the trainer will help caregivers complete one DICE session (describe behavior, investigate potential contributors, and create tailored strategies—the WeCareAdvisor Prescription). The trainer will encourage caregivers to try strategies and then use the tool to evaluate whether they were effective in managing the targeted behavior. Also, caregivers will be shown how to access other tool components.

For those randomized to receive telephone and email reminders (*n* = 81), trainers will contact caregivers once a week for the first month, and every other week between months 1 and 3 (total telephone contact from baseline to 3 months = 8 calls). Prior to calls, interviewers will examine the online dashboard to determine if caregivers are using the tool and if so, which components. Using a guided script (developed in our pilot), interviewers will remind caregivers to use the tool and complete weekly online symptom tracking measures, troubleshoot technical difficulties, and ask if caregivers have specific questions about their DICE sessions. The content of calls will be confined to tool use versus addressing clinical questions, or providing referrals, education, or psychosocial support. It is anticipated that each call will be approximately 10 min. This group will also receive email reminders once weekly (12 total) which prompts tool use. Then, between 3 and 6 months, participants randomized to this group will not receive any telephone or email reminders to evaluate if a “habit” has been formed such that tool use is sustained and independent of reminders. Based on pilot data, it is anticipated that the tool will be used about twice weekly during the trial.

The initial treatment group assigned to receive email reminders only (*n* = 82) will be prompted by a weekly email to use the tool between baseline and 3 months (12 total). As with the telephone prompts, email prompts will encourage caregivers to interact with the tool to address new behavioral events and to assess and manage previous or on-going BPSD. Between 3 and 6 months, this group will not receive email reminders. This will enable researchers to evaluate whether caregivers need ongoing personal prompts to use the tool, whether automated emails are sufficient prompts, and whether from 3 to 6 months a habit is formed such that the prompt type is not necessary.

WeCareAdvisor is the first online tool to operationalize DICE by posing a series of tested questions for each of its steps that caregivers can quickly complete and which lead to the generation of a customized treatment plan. For the Create step, > 1000 strategies were identified from previous clinical trials and literature reviews. Strategies were then matched (“tagged”) to different constellations of behaviors, caregiver, and PLWD-contexts and conditions. For example, sudden onset of agitated-type behavior is linked to the strategy of seeking medical help to evaluate medications, pain, and/or detection of underlying infection. This tailoring, unique to WeCareAdvisor, involves a process by which information about a person shared by the caregiver shapes content and format of messages provided in what is referred to as the “WeCareAdvisor Prescription.” Tailoring is shown in previous studies to improve outcomes for people with chronic illnesses (Enderlin et al., 2007). Caregivers can generate a customized WeCareAdvisor Prescription by engaging in the DICE approach, or alternately, caregivers can examine all > 1000 strategies listed in the Caregiver Survival Guide section of the tool (Kales et al., 2018) (Table 1).

**Table 1:**
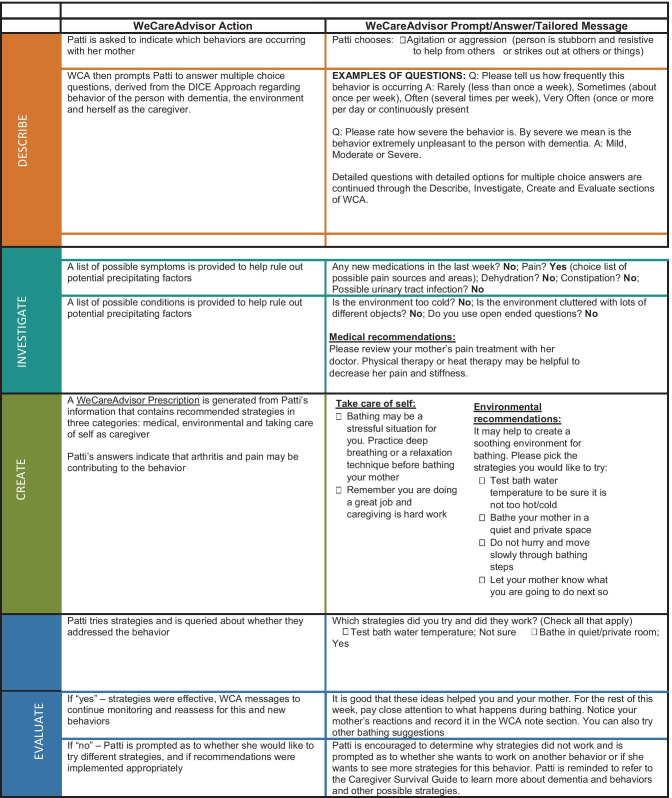
Hypothetical case example of DICE approach and tailoring strategies

### Waitlist Control Group

Participants randomized to the waitlist control group will receive access to the tool at the conclusion of their 3-month interview. Similar to the above, those assigned to telephone plus email prompts will receive 8 calls and 12 weekly emails during months 3–6; those assigned to receive only email prompts will receive 12 email reminders in that same time period. For all participants, regardless of whether they are in the initial treatment group or the waitlist control group, at the 6-month interview, trainers will instruct caregivers to remove the tool from their devices.

### Developing Habits to Use WeCareAdvisor

The WeCareAdvisor is designed as an aid to help caregivers address unmet needs on demand for disease education and knowledge about strategies to manage BPSD. Of importance is understanding whether use of WeCareAdvisor becomes part of the caregiver’s tool kit for managing dementia or a “habit” such that caregivers integrate tool use as part of their care routines. A habit is formally defined as an automatic response (requiring little thought) formed through repetitive actions (Kaushal & Rhodes, 2015; Lally et al., 2010; Stawarz et al., 2015). Habits are acquired through incremental strengthening association between a situation (cue, e.g., symptom) and an action (e.g., using DICE or other tool components) (Lally et al., 2010). Event-based cues such as receiving reminders have been shown to be effective in aiding habit formation (Stawarz et al., 2015). When desired behaviors are incorporated into a situation in which salient cues occur, habits are more likely to form. Another element promoting habit formation is making the desired behavior (tool use) easy to learn and receiving self-satisfaction (behaviors are managed) and the support and encouragement from others (reminders to use tool; daily care tips). Although it is unclear as to the length of time needed to form a habit, some studies suggest habit formation may take a median of 66 days (Kaushal & Rhodes, 2015). Early repetitions of the desired behavior have been shown to result in larger increases in automaticity (Brouwer et al., 2011; Enderlin et al., 2007; McClure et al., 2013). As such, this trial will evaluate whether different communications varying in intensity (high-touch through weekly telephone plus email vs. low-touch through automated weekly email reminders only) increase tool utilization. Both approaches have been shown to increase the number of logins and repeat visits to websites in other studies. Our proposed study design will enable researchers to determine the extent to which each form of contact contributes to outcomes.

### Data Collection

Data will be collected via telephone at each testing occasion: screen, baseline, 1-, 3-, and 6-month interviews. Caregivers can also choose to complete assessments in one phone interview (up to 90 min) or in segments over a 2-week timeframe. The baseline interview involves self-reported data about the caregiver’s health and wellbeing and caregiver-reported information on PLWD’s health, daily functional status and care needs, and behavioral symptom profile. Follow-up interviews at 1, 3, and 6 months collect primary outcome data.

Additionally, the WeCareAdvisor dashboard will capture the frequency of caregiver logins and use of individual components (DICE steps, Caregiver Survival Guide, daily tips, stress ratings).

Finally, following study completion, a study team member other than the interviewer or trainer will conduct a 15-min telephone survey to assess the caregiver’s experience as a study participant and using WeCareAdvisor.

### Randomization

Randomization will be stratified by relationship of caregiver (spouse/non-spouse) to PLWD to ensure group balance. Within each stratum, investigators will use a permuted block design to control for possible changes over time in participant mix and eliminate selection biases. Participants will be randomized as though the design were a 2 × 2 factorial, with caregivers randomized to one of four groups: treatment/high-intensity prompt, treatment/low-intensity prompt, waitlist control/high-intensity prompt, and waitlist control/low-intensity prompt.

### Power Analysis

Sample size has been calculated based on the following assumptions: (a) three primary outcomes at 3 months (caregiver distress with behaviors, caregiver confidence managing BPSD, and frequency/severity of BPSD), (b) treatment effect sizes for outcomes from the pilot and other nonpharmacologic trials (effect sizes for baseline adjusted changes are 0.35 for behavioral symptoms; 0.68 for caregiver distress; and 0.91 for caregiver confidence), (c) ability to detect a near medium effect size of 0.40 (moderate, nontrivial effect) similar to that observed in the pilot for BPSD, (d) a type I error rate of 0.017 (Bonferroni correction), (e) 80% power, and (f) 1:1 randomization. Based on these assumptions, 260 caregivers are needed (130 per group) to attain 80% power using a two-sample *t*-test with a two-sided comparison of the two treatment groups on the main trial endpoint at 3 months. As a 20% attrition rate is anticipated by 3 months based on previous trials, the study will enroll 326 caregivers. Power for comparing prompt intensity is similar. Sample size calculations for exploratory analyses have not been conducted, but sufficient power is available to support these analyses as they are across the entire study sample. For mediation analyses, a sample size of over 200 is sufficient to detect statistical mediation with 80% power using the Sobel method as long as both legs of the mediated effect have standardized effect sizes of at least 0.26, considered halfway between “small” and “medium” effect sizes.

### Data Analysis

Data from all caregivers randomized to the initial treatment group will be part of the primary analyses regardless of actual engagement level with the tool (intention to treat (ITT) analysis), so that each caregiver will be included in the analysis in the group to which they are randomized. A repeated measures analysis of variance (ANOVA) will be used to assess differences over time, with the primary test that of the interaction between treatment group and time. Covariates of interest might include comorbidities, number/type of medications of persons living with dementia, caregiver relationship, gender of persons living with dementia, and caregiver although we anticipate that randomization will balance the factors across groups, eliminating the need to include them in the analysis. Further, all models will adjust for relationship of caregiver to person living with dementia (stratification variable). Repeated measures ANOVA provides unbiased estimates of intervention effects given that the data are missing at random. However, should the degree of missing data be large, multiple imputation models will be used in which missingness is modeled as a function of covariates, to impute missing outcomes.

Utilization patterns and predictors of use of WeCareAdvisor will also be assessed. Dashboard data will be examined to determine the number of completed DICE sessions from baseline to 3 months and from 3 to 6 months. Effects of high- and low-intensity prompts on the three primary outcomes from baseline to 3 months will be compared using a similar repeated measures ANOVA model to that described above, now including the prompt level intensity in the model, averaging over group to combine the first 3 months in the treatment group with the second 3 months in the control (their active period). Analyses of predictors of utilization are important to identify to understand which caregivers are most likely to use the tool and DICE steps, and to help modify the platform for those who may not use the tool.

Exploratory analyses will evaluate the impact of WeCareAdvisor on psychotropic medication use, functional dependence, and other outcomes of interest. Possible intervention moderator effects by caregiver gender, race (nonwhite/white), and relationship (spouse/nonspouse) will be examined. Mediation modeling will be conducted using structural equation modeling techniques to determine what proportion of WeCareAdvisor’s impact on primary outcomes can be explained by its impact on change in theoretically derived mediating variables (self-efficacy, readiness). Methods for testing intervention effects in the context of randomized trials with multiple waves of mediator and outcome data will be implemented.

## Impact of COVID-19 on Trial Design

Although aims, hypotheses, outcomes, and other basic study design features remain intact, COVID-19 has necessitated critical modifications summarized in Table 2.

**Table 2:** Study design changes due to COVID-19 pandemic

Design element	Pre-COVID-19	COVID-19 design	Rationale
Eligibility criteria	1. Enrollment of caregivers and people living with dementia 2. No eligibility requirement concerning internet access as it was to be provided by study 3. Live with or within 5 miles of person living with dementia	1. Enrollment of caregivers only 2. No clinical data from person living with dementia collected 2. Must have own access to internet via smart phone, computer, or tablet 3. Eliminated requirement to live with or within 5 miles of person living with dementia	Criteria were broadened to be more inclusive of the dementia caregiver population nationally. Revised criteria allow enrollment of long distance caregivers if they are managing behavioral symptoms from afar. One limitation is that PLWD are no longer enrolled in the study to enable direct administration of cognitive status tests to confirm diagnosis and identify disease stage. Two caregiver report-based measures (AD8 and FAST) have been added to address this limitation
Recruitment	Restricted to Philadelphia and Delaware Valley Tri-State Area	1. Any geographic region in the USA 2. Stratified into US Government ten regions	To assure greater representation, a diversified sample will be enrolled that includes caregivers living in urban, suburban, and rural areas as well as throughout the country to support generalizability of findings. Outreach to rural areas will be emphasized given limited resources for dementia support in those areas
Cognitive status of person living with dementia	Administration of the Mini-mental status Examination in the home to the PLWD	Caregiver report on two scales to stage. The AD-8 (Galvin et al., 2005) is used at screening to inform eligibility. The Functional Assessment Staging Tool (Reisberg, 1988) is used in baseline and follow-up interviews to inform level of functioning	As per above, two scales with good psychometric properties will be used to determine disease stage through caregiver report
Interviews	Conducted in-person in caregiver and PLWD homes	Conducted via HIPAA-telephone line	As it is not feasible to conduct in-person visits due to COVID-19, HIPAA-compliant telephones will be used for interviews, providing greater access to caregivers across the country
Randomization	Single-blinded. Completed by interviewer at completion of the in-person baseline interview using an envelope system and also responsible for instructing caregiver in use of tool	1. Investigators and interviewers remain masked to group allocation 2. Randomization through RedCap 3. Caregiver informed of group allocation by a research staff member other than interviewer 4. Training in WeCareAdvisor occurs by research staff member other than interviewer 5. Caregivers will be asked to not inform interviewer of the group they were in	It is challenging to institute double blinding in nonpharmacological trials. However, these modifications achieve better blinding. Responsibilities are separated such that interviewers will remain blind throughout the study, and the interventionist who trains in tool use will only have that role. By asking caregivers to not disclose their group assignment may help further assure interviewers remain blind. Following conclusion of interviews, interviewers will be asked to guess group assignment and indicate a rationale for their guess
WeCare-Advisor platform	Available on preprogrammed iPad provided to caregivers by study with email notifications for daily tips and reminders	1. Expanded functionality such that tool will be available on any internet-capable device owned by caregiver (smartphone, computer, tablet) 2. Text or email notifications now possible 3. Text-to-speech functionality specifically to DICE section has been added to reduce literacy dependency	The change in tool functionality permits wider reach and participation of caregivers. It also gives caregivers a choice as to their preferred way of using the tool (by phone, tablet, or computer) enabling greater access and integration of the tool into daily care routines Also, the text-to-speech function will lessen the dependency on health literacy and enable caregivers to hear while they read making materials more accessible and strengthen learning
WeCare-Advisor content changes	Information provided about dementia, behavioral symptoms, taking care of self in a section of the tool referred to as Caregiver Survival Guide	Additional information about COVID-19, updated dementia information, and more details about involving people living with dementia in daily activities	With COVID-19, caregivers face new care challenges, including understanding the pandemic and how to protect themselves and the PLWD. Also, with more time spent at home, caregivers are experiencing greater social isolation and report having to manage more behavioral symptoms. Providing education on these issues is essential
Instruction in use of WeCare-Advisor	Interviewer provides caregiver with preprogrammed iPad; walks caregiver through using app in-person and one DICE approach for a targeted behavioral symptom	A member of the research team (not the interviewer) helps caregiver gain access to the online platform and walks caregiver through using app via telephone and one DICE approach for a targeted behavioral symptom. A brief instructional video will also be available for caregivers to refer to if they seek a refresher. Additionally, caregivers will have access to a 1–800 number to call project team if they are having technical difficulties	The approach reinforces instruction and support of caregivers

The most significant modification involves expanding the functionality of the WeCareAdvisor platform from operating only on a grant-issued iPad to being accessible through any caregiver owned device (smartphone, tablet, or computer). This, along with greater acceptability of use of telephone for health-care-related issues, has opened up the opportunity to expand recruitment from the Philadelphia region and in-person home-based interviews to a national recruitment strategy and use telephone interviewing.

Thus, another major modification concerns the recruitment strategies that will be deployed to enroll 326 caregivers. The original trial targeted a regional area and intended to use as its primary strategy direct mail inviting study participation that would be issued by local community-based agencies to their constituents. The strategies now will be national in scope, and an attempt will be made to assure representation across 10 regions (as designated by the US Department of Health and Human Services). These strategies include (a) applying Geographic Information System (GIS) technology to identify neighborhoods with older adults and ensure representation across sex, race, income, and education; (b) collaborating with aging service providers, community development centers, federally qualified health centers, and culturally specific organizations; (c) utilizing national and culturally specific clinical research registries; (d) writing letters to nationally syndicated advice columns; (e) leveraging professional contacts and national networks; (f) social media posts; and (g) asking participants to share information with other caregivers in their social circles. Additionally, a list of key service providers, community development centers, federally qualified health centers, and culturally specific organizations serving caregivers, older adults, and/or people living with dementia across the ten regions and will be mapped onto census tracts across the country using GIS technology to help prioritize and stage recruitment efforts.

### Eligibility

Eligibility criteria for this trial have not significantly changed from the original trial. Although people living with dementia will not be enrolled in this trial, eligibility criteria concern both the person living with dementia and the caregiver. Criteria concerning people living with dementia as reported by the caregiver include (a) diagnosis of dementia; (b) one or more dementia-related behaviors that is distressing or challenging to the caregiver; (c) if prescribed, on a stable dose (≥ 60 days) of antidementia or psychotropic medications; (d) responsive to surroundings and simple commands; and (e) scores 2 or higher on the AD8 cognitive screening test. As to the latter, this brief, caregiver-reported measure identifies changes in memory, new learning, financial management, and judgment over a period of several years (Galvin et al., 2005). A score of 2 or higher reflects probable cognitive impairment and is used as a cutoff for this study.

Criteria concerning caregivers include (a) being the primary caregiver for at least 6 months; (b) age 21 years or older; (c) no plans to place the person living with dementia in long-term care for the next 6 months; (d) has no visual or hearing impairments that limit ability to use the WeCareAdvisor or be interviewed; (e) has internet access and facileness in using a smartphone, tablet, or computer; and (f) is not participating in other studies to evaluate a supportive or education intervention. Both the person living with dementia and/or caregiver also must not have been hospitalized three or more times over the past year, have a terminal disease, or be in active treatment for cancer.

## Discussion

WeCareAdvisor addresses a critical public health priority to support family caregivers in managing BPSD, a common dementia-related clinical symptom with significant negative impacts. With COVID-19, the need to manage BPSD has been exacerbated by having to stay at home, practice social distancing, mask wearing, limited access to supportive services, social isolation, and disruption of daily routines.

WeCareAdvisor provides an evidence-informed, innovative, nonpharmacological option for caregivers. Training in use of the tool is minimally burdensome; it delivers on demand vetted disease education and through its interactive online platform, generates strategies specific to the circumstances detailed by caregivers. This Phase III efficacy trial will evaluate whether use of the WeCareAdvisor lowers caregiver stress and increases their confidence and the effects of different types of reminders or prompts on tool use and outcomes. Understanding if caregivers require prompts and if so what type, is an underexplored area of research in technology use and one of the unique contributions of this trial that can directly inform dissemination, scaling, and commercialization.

Evidence suggests that providing knowledge and skills to caregivers via web-based online formats is effective (Hattink et al., 2015; Kwok et al., 2014; Lorig et al., 2012). By enabling families to access WeCareAdvisor via smart phone technology may also address the persistent disparity in access to broadband internet between African American and White caregivers and families with limited resources (Anderson, 2015; Smith, 2015; Ryan, 2017).

This study protocol is innovative in important ways. First, an online interactive program to help families manage one of the most stressful symptoms of dementia (BPSD) has not been systematically developed or tested previously nationally. Second, WeCareAdvisor has noteworthy and novel features. It is the first to be grounded in a conceptual model for understanding the etiology and phenotype of BPSD. It is the first to operationalize an algorithmic approach to tailor strategies to symptom presentation. The tool can be used by caregivers of persons with any disease etiology and stage and behavioral presentation, making it relevant to all families and with disease progression. It does not require caregivers, who are typically pressed for time, to complete training sessions over the course of weeks or months as in individual or group interventions to learn behavioral management strategies (Werner et al., 2017). Additionally, caregivers can use the tool and navigate through any of its components based on their preference, need, and time, putting caregivers in control. Third, as an online tool, a health professional is not needed for its use. Thus, its cost would be minimal and the potential for dissemination, scalability, and reach is high. Fourth, the study design is innovative in its dual focus on tool efficacy and utilization. Finally, COVID-19 presents a unique opportunity to enhance design features although study aims, hypotheses, randomization, and group allocation remain intact. The changes in design, while initially challenging, affords important opportunities to test WeCareAdvisor with a national sample of family caregivers that is more diverse geographically, racially, and ethnically. The enhanced design features will yield new knowledge concerning tool efficacy on key outcomes of importance to families, and whether prompting strategies are needed to reinforce continuous tool use, as well as whether certain caregivers benefit more than others. These study findings will be essential to future dissemination efforts of WeCareAdvisor and guide a pathway towards commercialization.

In conclusion, lessons learned from this trial about efficacy, adoption, and utilization may shorten the time frame of the classic 17-plus year gap from idea inception to widespread use of evidence and need for a translational phase that adopts the tool for implementation by health care systems (Gitlin & Czaja, 2016; Institute of Medicine, 2013; Hodgson & Gitlin, 2021). Moreover, the study design enables an evaluation of best practices for designing national recruitment strategies to enroll caregivers of people living with dementia, setting the stage for widespread dissemination and commercialization opportunities.

## Declarations

### Compliance with Confidentiality

All work has or will be conducted in compliance with confidentiality (HIPAA) standards.

## References

[CR1] AARP. (2016). *Caregivers & technology: What they want and need*. Retrieved from: http://www.aarp.org/content/dam/aarp/home-and-family/personaltechnology/2016/04/Caregivers-and-Technology-AARP.pdf

[CR2] Alzheimer’s Association.  (2020). 2020 Alzheimer’s disease facts and figures. Alzheimer's and Dementia.

[CR3] Anderson, M. (2015). Racial and ethnic differences in how people use mobile technology. *Pew Research Center.* Retrieved from http://www.pewresearch.org/facttank/2015/04/30/racial-and-ethnic-differences-in-how-people-use-mobile-technology/

[CR4] Arthur PB, Gitlin LN, Kairalla JA, Mann WC (2017). Relationship between the number of behavioral symptoms in dementia and caregiver distress: What is the tipping point?. International Psychogeriatrics.

[CR5] Beeri M, Werner P, Davidson M, Noy S (2002). The cost of behavioral and psychological symptoms of dementia (BPSD) in community dwelling Alzheimer’s disease patients. International Journal of Geriatric Psychiatry.

[CR6] Black BS, Johnston D, Rabins PV, Morrison A, Lyketsos C, Samus QM (2013). Unmet needs of community-residing persons with dementia and their informal caregivers: Findings from the maximizing Independence at home study. Journal of the American Geriatrics Society..

[CR7] Brouwer W, Kroeze W, Crutzen R, De Nooijer J, De Vries NK, Brug J, Oenema A (2011). Which intervention characteristics are related to more exposure to internet delivered healthy lifestyle promotion interventions? A systematic review. Journal of Medical Internet Research.

[CR8] Cuffalo L, Di Lorenzo F, Bonavita S, Tedeschi G, Leocani L, Lovorgna L (2020). Dementia care and COVID-19 pandemic: A necessary digital revolution. Neurological Sciences.

[CR9] Czaja SJ, Charness N, Fisk AD, Hertzog C, Nair SN, Rogers WA, Sharit J (2006). Factors predicting the use of technology: Findings from the Center for Research and Education on Aging and Technology Enhancement (CREATE). Psychology and Aging.

[CR10] Dang, S., Nedd, N., Nair, S., Roos, B., & Al, E. (2004). Utilization of TLC technology by dementia family caregivers. *The Gerontologist*, *44*(1), 604. Retrieved from: http://search.proquest.com/docview/61343408?accountid=14643

[CR11] De Vugt ME, Stevens F, Aalten P, Lousberg R, Jaspers N, Winkens I, Verhey FRJ (2004). Do caregiver management strategies influence patient behaviour in dementia?. International Journal of Geriatric Psychiatry.

[CR12] Enderlin C, Richards K, Beck C, McSweeney JC, Jones TC, Roberson PK (2007). Tailored biobehavioral interventions: A literature review and synthesis. Research and Theory for Nursing Practice.

[CR13] Forrester SN, Gallo JJ, Smith GS, Leoutsakos JMS (2016). Patterns of neuropsychiatric symptoms in mild cognitive impairment and risk of dementia. The American Journal of Geriatric Psychiatry: Official Journal of the American Association for Geriatric Psychiatry.

[CR14] Fox, S., Duggan, M. & Purcell, K. (2013). Family caregivers are wired for health. *Pew Research Center*. Retrieved from: https://www.pewresearch.org/internet/2013/06/20/family-caregivers-are-wired-for-health/2013/06/20/family-caregivers-are-wired-for-health/

[CR15] Galvin JE, Roe CM, Powlishta KK, Coats MA, Much SJ, Grant E, Miller JP, Storandt M, Morris JC (2005). The AD8: A brief informant interview to detect dementia. Neurology.

[CR16] Gitlin LN, Kales HC, Lyketsos CG (2012). Nonpharmacologic management of behavioral symptoms in dementia. The Journal of the American Medical Association.

[CR17] Gitlin LN, Czaja SJ (2016). Behavioral intervention research: Designing, evaluating, and implementing.

[CR18] Hattink B, Meiland F, Van Der Roest H, Kevern P, Abiuso F, Bengtsson J, Droes RM (2015). Web-based STAR e-learning course increases empathy and understanding in dementia caregivers: Results from a randomized controlled trial in the Netherlands and the United Kingdom. Journal of Medical Internet Research.

[CR19] Hodgson N, Gitlin LN, Winter L, Hauck WW (2014). Caregiver’s perceptions of the relationship of pain to behavioral and psychiatric symptoms in older community-residing adults with dementia. The Clinical Journal of Pain.

[CR20] Hodgson, N. & Gitlin, L. N. (2021). Implementing and sustaining family care programs in real world settings: Barriers and facilitators. In J. Gaugler (Ed.). *Bridging the Family Care Gap*. Philadelphia: Elsevier. Chapter 17, pp. 305–354.

[CR21] Institute of Medicine (2013). The CTSA Program at NIH: Opportunities for advancing clinical and translational research.

[CR22] Jutkowitz E, Kuntz KM, Dowd B, Gaugler JE, MacLehose RF, Kane RL (2017). Effects of cognition, function, and behavioral and psychological symptoms on out-of-pocket medical and nursing home expenditures and time spent caregiving for persons with dementia. Alzheimer’s and Dementia.

[CR23] Kales HC, Kim HM, Zivin K, Valenstein M, Seyfried LS, Chiang C, Blow FC (2012). Risk of mortality among individual antipsychotics in patients with dementia. American Journal of Psychiatry.

[CR24] Kales, H. C., Gitlin, L. N., & Lyketsos, C. G. (2014). The time is now to address behavioral symptoms of dementia. *Generations*, *38*(3), 86–95. Retrieved from: https://www.researchgate.net/publication/272090822_The_Time_is_Now_to_Address_Behavioral_Symptoms_of_Dementia

[CR25] Kales, H. C., Gitlin, L. N., Stanislawski, B., Mrya Kim, H., Marx, K., Turnwald, M., …, & Lyketsos, C. G. (2018).Effect of the WeCareAdvisor^TM^ on family caregiver outcomes in dementia: A pilot randomized control trial. *BMC Geriatrics, 18(113).*10.1186/s12877-018-0801-8 10.1186/s12877-018-0801-8 PMC5946471 29747583

[CR26] Kaushal N, Rhodes RE (2015). Exercise habit formation in new gym members: A longitudinal study. Journal of Behavioral Medicine.

[CR27] Kwok T, Au A, Wong B, Ip I, Mak V, Ho F (2014). Effectiveness of online cognitive behavioral therapy on family caregivers of people with dementia. Clinical Interventions in Aging.

[CR28] Lally P, Van Jaarsveld CHM, Potts HWW, Wardle J (2010). How are habits formed: Modelling habit formation in the real world. European Journal of Social Psychology.

[CR29] Lawlor B (2002). Managing behavioural and psychological symptoms in dementia. The British Journal of Psychiatry.

[CR30] Lorig K, Thompson-Gallagher D, Traylor L, Ritter PL, Laurent DD, Plant K, Hahn TJ (2012). Building better caregivers. Journal of Applied Gerontology.

[CR31] Malani, P., Kullgreen, J., Solway, E., Piette, J., Singer, D. & Kirch, M. (September 2020). *National poll on healthy aging: Loneliness among older adults before and during the COVID-19 pandemic.* Retrieved from: https://deepblue.lib.umich.edu/bitstream/handle/2027.42/162549/0212_NPHA-loneliness-report-FINAL-09112020-handle.pdf?sequence=5&isAllowed=y

[CR32] Maust DT, Kim HM, Seyfried LS, Chiang C, Kavanagh J, Schneider LS, Kales HC (2015). Antipsychotics, other psychotropics, and the risk of death in patients with dementia: Number needed to harm. JAMA Psychiatry.

[CR33] McClure JB, Shortreed SM, Bogart A, Derry H, Riggs K, John JS, An L (2013). The effect of program design on engagement with an internet-based smoking intervention: Randomized factorial trial. Journal of Medical Internet Research.

[CR34] National Alliance for Caregiving. (2011). *E-connected family caregiver: Bringing caregiving into the 21st century*. Retrieved from: https://www.caregiving.org/wp-content/uploads/2020/05/FINAL_eConnected_Family_Caregiver_Study_Jan-2011.pdf

[CR35] Rabins P, Lyketsos C, Steele C (2006). Practical dementia care.

[CR36] Regier NG, Hodgson NA, Gitlin LN (2020). Neuropsychiatric symptom profiles of community-dwelling persons living with dementia: Factor structures revisited. International Journal of Psychiatry.

[CR37] Reisberg B (1988). Functional assessment staging (FAST). Psychopharmacology Bulletin.

[CR38] Ryan, C. (2017). Computer and Internet Use in the United States: 2016. *American Community Survey Reports*. Retrieved from: https://www.census.gov/content/dam/Census/library/publications/2018/acs/ACS-39.pdf

[CR39] Sacuiu S, Insel PS, Mueller S, Tosun D, Mattsson N, Jack CR, Scott MacKin R (2016). Chronic depressive symptomatology in mild cognitive impairment is associated with frontal atrophy rate which hastens conversion to Alzheimer dementia. American Journal of Geriatric Psychiatry.

[CR40] Salzman, C., Jeste, D. V., Meyer, R. E., Cohen-Mansfield, J., Cummings, J., Grossberg, G. T., … Zubenko, G. S. (2008). Elderly patients with dementia-related symptoms of severe agitation and aggression: Consensus statement on treatment options, clinical trials methodology, and policy. In *Journal of Clinical Psychiatry* (Vol. 69, pp. 889–898). 10.4088/JCP.v69n0602 10.4088/jcp.v69n0602 PMC2674239 18494535

[CR41] Schneider LS, Tariot PN, Dagerman KS, Davis SM, Hsiao JK, Ismail MS, Lieberman JA (2006). Effectiveness of atypical antipsychotic drugs in patients with Alzheimer’s disease. New England Journal of Medicine.

[CR42] Smith, A. (2015). U.S. smartphone use in 2015. *Pew Research Center*. Retrieved from: https://www.pewresearch.org/internet/2015/04/01/us-smartphone-use-in-2015/

[CR43] Stawarz, K., Cox, A. L., & Blandford, A. (2015). Beyond self-tracking and reminders : Designing smartphone apps that support habit formation. *Chi*, 2653–2662. 10.1145/2702123.2702230

[CR44] UsAgainstAlzheimers. (2020). UsAgainstAlzheimer’s survey on COVID-19 and Alzheimer’s community summary of findings for June 2020 survey (Survey #4). Retrieved from: https://www.usagainstalzheimers.org/sites/default/files/2020-06/UsA2%20COVID%20Survey%204%20Summary%206.25.20.pdf

[CR45] Van Den Wijngaart MAG, Vernooij-Dassen MJFJ, Felling AJA (2007). The influence of stressors, appraisal and personal conditions on the burden of spousal caregivers of persons with dementia. Aging & Mental Health.

[CR46] Werner NE, Stanislawski B, Marx KA, Watkins DC, Kobayashi M, Kales H, Gitlin LN (2017). Getting what they need when they need it. Applied Clinical Informatics.

[CR47] Werntz AJ, Dodson CS, Schiller AJ, Middlebrooks CD, Phipps E (2015). Mental health in rural caregivers of persons with dementia. SAGE Open.

